# Molecules important for thyroid hormone synthesis and action - known facts and future perspectives

**DOI:** 10.1186/1756-6614-4-S1-S9

**Published:** 2011-08-03

**Authors:** Klaudia Brix, Dagmar Führer, Heike Biebermann

**Affiliations:** 1 School of Engineering and Science, Jacobs University Bremen, Research II, Campus Ring 6, 28759 Bremen, Germany; 2 Universitätsklinikum Leipzig Medizinische Klinik III, 04103 Leipzig, Germany; as of June 2011: Klinik für Endokrinologie, Zentrum für Innere Medizin, Bereich Forschung und Lehre im Zentrallabor, 45147 Essen, Germany; 3 Institute of Experimental Pediatric Endocrinology, Charité Universitätsmedizin Berlin, Augustenburger Platz 1, 13353 Berlin, Germany

## Abstract

Thyroid hormones are of crucial importance for the functioning of nearly every organ. Remarkably, disturbances of thyroid hormone synthesis and function are among the most common endocrine disorders affecting approximately one third of the working German population. Over the last ten years our understanding of biosynthesis and functioning of these hormones has increased tremendously. This includes the identification of proteins involved in thyroid hormone biosynthesis like Thox2 and Dehal where mutations in these genes are responsible for certain degrees of hypothyroidism. One of the most important findings was the identification of a specific transporter for triiodothyronine (T3), the monocarboxylate transporter 8 (MCT8) responsible for directed transport of T3 into target cells and for export of thyroid hormones out of thyroid epithelial cells. Genetic disturbances of MCT8 in patients result in a biochemical constellation of high T3 levels in combination with low or normal TSH and thyroxine levels leading to a new syndrome of severe X-linked mental retardation. Importantly mice lacking MCT8 presented only with a mild phenotype, indicating that compensatory mechanisms exist in mice. Moreover, it has become clear that not only genomic actions of T3 exist. T3 is also capable to activate adhesion receptors and it signals via activation of PI3K and MAPK pathways. Most recently, thyroid hormone derivatives were identified, the thyronamines which are decarboxylated thyroid hormones initiating physiological actions like lowering body temperature and heart rate, thereby acting in opposite direction to the classical thyroid hormones. So far it is believed that thyronamines function via the activation of a G-protein coupled receptor, TAAR1. The objective of this review is to summarise the recent findings in thyroid hormone synthesis and action and to discuss their implications for diagnosis of thyroid disease and for treatment of patients.

## Introduction

## Health consequences of disturbed thyroid hormone action

Thyroid hormones are of significant importance for regular functioning of almost all body organs. Thyroxine (T4) is the main hormone released from the thyroid gland and it is transformed into biologically active 3',3,5-triiodothyronine (T3) via 5'-deiodinases of thyroid hormone target cells [1]. For thyroid hormone synthesis, sufficient supply of the thyroid gland with essential micronutrients such as iodine and selenium is crucial.

The most important target tissues of thyroid hormones are the central nervous system, the cardiovascular system and the skeleton. Moreover, due to the increasing incidence of obesity worldwide, thyroid hormone action in adipose tissue has re-gained increasing interest for understanding energy homeostasis [2].

Disturbance of thyroid hormone synthesis and thyroid hormone actions, further referred to as thyroid disorders, are among the most common endocrine afflictions and they require special attention in specific life phases. For instance, undiagnosed hypothyroidism during pregnancy will lead to irreparable central nervous system (CNS) defects in the newborn because the development of the child *in utero* is critically affected by the mother’s thyroid status [3]. Therefore and to avoid fatal consequences of a lack of thyroid hormone synthesis after birth, eventually leading to severe mental retardation, thyroid stimulating hormone (TSH)-screening of newborns [4] was implemented in Germany and in other countries. In action since the late 1970s and with a clear improvement in those areas of the world where TSH-screening was first introduced, a marked decrease in cretinism was observed.

Thyroid hormone (TH) action serves important regulatory functions throughout all phases of life. Disturbed TH action is linked with major health problems especially in critical life phases such as development, disease or ageing. Thus, lack of TH action in the adult brain causes impaired neuro-cognitive function and psychiatric states such as severe depression and dementia [5]. Not only hypothyroidism but also hyperthyroidism affects the CNS and frequently results in agitation, increased irritability and dysregulation of body temperature. There is ample epidemiological evidence that both, hyper- and hypothyroidism confer an increased risk for cardiovascular morbidity (e.g. arrhythmia, heart failure and stroke) and mortality. Moreover, thyroid disorders affect maturation and turnover rates of bone. For example, children with hyperthyroidism show advanced bone age, and post-menopausal women with thyrotoxicosis are at risk for osteoporosis. Furthermore, adiposity is associated with altered levels of measurable thyroid function parameters, whereby it is not known however whether the obesity state influences the set-point for thyroid function parameters or vice versa. Besides the common health issues, life threatening conditions may either arise from severely disturbed thyroid hormone action or are accompanied by altered thyroid hormone action. These include e.g. thyroid storm with a mortality of more than 30%, which at present can only be diagnosed on clinical grounds since measurement of thyroid hormone levels is not useful and may be identical to those in patients with uncomplicated thyrotoxicosis or the non-thyroid illness syndrome in intensive care patients with e.g. sepsis.

Health-economical dimensions of thyroid disorders are shown most plainly by the example of latent hypothyroidism which is evident in about 20% of all individuals at the age of 60-79 years, with higher incidences in women than in men [6]. On top of this, an increase in the number of patients with thyroid disorders is expected in the future. Possible causes are anthropogenic in origin and are represented by a variety of environmental substances, so-called endocrine disruptors such as estrogen-derivates and PBCBs, that affect the endocrine system at large including the thyroid gland and thyroid hormone actions [7].

Furthermore, classical epidemiology on large cohorts (SHIP and KORA) has underlined that manifest and even more so, subclinical thyroid disorders are often associated with other, quite frequently occurring pathophysiological processes [8,9]. These include, but are not limited to left-ventricular hypertrophy, atherosclerosis with increased risk for stroke and coronary heart disease, disorders in lipid metabolism, osteoporosis and neurodegenerative diseases. However, subclinical hyperthyroidism was found not to be associated with e.g. changes in blood pressure in the SHIP cohort study [9] although serum TSH levels in the upper reference range were indicative of endothelial dysfunction [10], highlighting the necessity of further investigations aiming at a better definition of the healthy thyroid state.

## New aspects of thyroid hormone synthesis

A number of molecular causes of thyroid dysgenesis, accounting for approx. 80% of cases suffering from congenital hypothyroidism (CH), have been identified over the last few years and were shown to include mutations in transcription factors important for thyroid development such as *Pax8*, *Nkx2.1*, *FoxE1*, and *HoxA3*[11]. Mutations in these genes often also lead to developmental dysfunction in organs other than the thyroid, thereby affecting the lung and the central nervous system (*Nkx2.1*) [12], or the kidney (*PAX8*) [13]. However, only about 5% of these genetic defects could account for the pathogenesis of thyroid dysgenesis [14]. Therefore, non-classical mechanisms involving epigenetic regulation are to be considered in future as modulators of gene expression during development of the thyroid.

In addition, genetic defects have been detected in all those genes bearing an essential role in the biosynthesis of thyroid hormones (approx. 10 to 15% of CH cases). These include the gene for the prohormone thyroglobulin (Tg) itself [15]; the thyroid stimulating hormone receptor (*TSHR*) [16], and the thyroid peroxidase (*TPO*) [17]; as well as genes relevant for iodide transport, i.e. the sodium-iodide symporter (*NIS*) [18] at the basolateral and *Pendrin*[19] at the apical pole of thyroid epithelial cells. Furthermore, the gene products of *Dehal*[20] and *Thox2*[21] as well as inactivating and activating mutations of the Gs alpha gene [22] have been added to the growing list of candidate proteins with important thyroid-specific functions. Recently, potassium channel subunits *Kcnq1* and *Kcne2* were found to be crucial for TSH-stimulated thyroid hormone biosynthesis. Mice lacking *Kcne2* suffer from impaired iodide uptake into the thyroid gland and exhibit signs of hypothyroidism [23].

A more profound understanding of thyroid hormone biosynthesis has also led to a clearer view on the molecular regulation of the thyroid gland and has increased our knowledge about the differentiated state of thyroid epithelial cells. In this context, novel players have been identified, which are important for the maintenance of thyroid physiology, i.e. molybdenum-dependent enzymes with important functions in the oxidative system of thyrocytes [24] and molecules that play a role in thyroglobulin processing for thyroid hormone liberation and release from the thyroid gland (megalin and cysteine cathepsins) [25-27].

Many of these players have been known for long, but it was only recently that the impact of mutations in thyroid-specific genes for thyroid physiology was set into context for a clearer depiction of the interactions and cross-relations between the many molecules and pathways relevant for thyroid biology. The regulation governing the functions of thyroid-specific proteins and their precise roles in the etiology of thyroid disorders will enable us to understand their mechanisms of action in detail and will advance our knowledge on the molecular biology of the thyroid gland.

## Novel concepts of transmembrane and intracellular thyroid hormone actions

**Figure 1:**
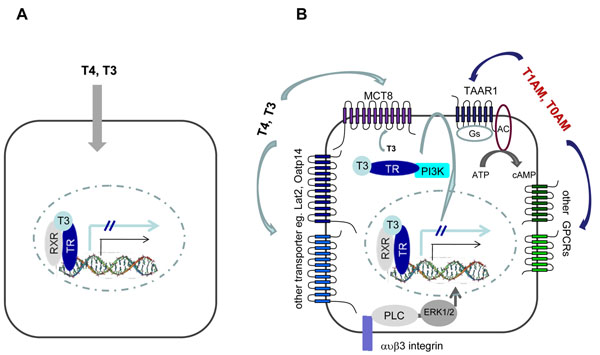
**Old and new concepts of thyroid hormone action. A: Old concept of thyroid hormone action.** In former times it was assumed that thyroid hormones are able to pass the plasma membrane by passive transport. Once in the cytosol T4 is deiodinated to T3 which exerts genomic effects by binding to the thyroid hormone receptor (TR). After hetero-dimerization with other nuclear receptors like retinoic X receptor (RXR), transcriptional regulation is initiated resulting in activation or inactivation of target genes. **B: New concepts of thyroid hormone action.** Thyroid hormones enter a target cell via specific transporters, e.g. T3 uses the monocarboxylate transporter MCT8 while T4 entry is mediated by Lat2 or Oatp14. Moreover, T3 can interact with α*v*β3 integrins to induce ERK1/2 signalling. Cytosolic T3 exerts genomic effects but can additionally also act by non-genomic means after TR binding and activation of down-stream PI-3 kinase. Likewise, the naturally occurring iodothyronine T2 is believed to stimulate metabolic rates via mitochondrial pathways, thereby bypassing genomic regulation. Besides thyroid hormones, derivatives like the thyronamines T1AM or T0AM, modulate the action of T3, e.g. counter-acting its effects in certain target cells. Thyronamines (TAMs) bind to and activate G-protein coupled receptors (GPCRs) of the trace amine associated receptor (TAAR) family. So far, it is only known that TAAR1 is activated by TAMs and signals via adenylylcyclase (AC) activation with subsequent rise of cAMP levels. However other GPCRs are likely targets for thyroid hormone derivatives.

Previous textbook knowledge of thyroid hormone entry into cells included a diffusion-based passive transmembrane passage for thyroid hormones which are lipophilic but charged molecules. However, an eye-opening revelation was the identification of the MCT8 transporter (*SLC16A2*) that selectively enables T3 transport into target cells [28]. This important finding explained at the molecular level the phenotype of severe X-linked mental retardation and disrupted locomotor development of patients with the Allan Herndon Dudley (AHD) syndrome, already described in the 1940s. These patients carry a defect in the MCT8 transporter, which explains why T3 does not reach all of its target cells in sufficient quantities during development. At the same time AHD patients show highly elevated serum T3 levels with unaltered to elevated TSH contradicting the “normal feedback scenario”. Thus it needs to be investigated further whether the abnormally high T3 levels in conditions of MCT8 defects that are linked with normal to slightly elevated TSH levels may cause thyrotoxic effects in TH target tissues other than the central nervous system [29]. However this clinical observation prompted thyroidologists to question the classical views on thyroid regulation. Subsequently, other TH transporter candidates have been identified (e.g. Oatp14, Lat1, Lat2, Mct10) in mice and require functional characterization [30-33]. It will be important in future to not only identify the tissue distribution patterns of TH transporters but more so, to clarify their potential contributions to the intracellular compartmentalisation of TH so that a clearer picture is gained on the sub-cellular gradients of TH and how specific compartments within cells, besides the nucleus, are supplied with TH in order to contribute or to withstand non-classical TH actions. Further TH transporters are eagerly awaited to be defined (Figure 1).

Another unexpected finding of recent research projects was the identification of thyroid hormone derivatives, the so-called thyronamines [34]. These are decarboxylated thyroid hormones that exert effects with kinetics different from those of thyroid hormone mediated actions and principally counter-acting them (so called “cool thyroid hormones”). Most obvious effects of thyronamines are observed in decreased heart rates and in negative regulation of body temperature. Thyronamines display their effects via activating a new subfamily of G-protein-coupled receptors (GPCRs), the *trace amine associated receptors* (TAARs) [35-37]. Because of their central roles in signalling resulting in a multitude of regulatory effects on almost all biological processes, GPCRs are interesting targets for pharmacological intervention [38]. Hence, the development of new therapeutic agents for regulation of heart beat frequency and of body temperature is now discussed in light of emergency treatments of stroke or acute myocardial infarction. Moreover, thyronamine-based therapies are expected to enter intensive care medical treatments.

Furthermore several lines of evidence suggest that in addition to the classic nuclear thyroid hormone action, rapid plasma membrane-initiated effects of thyroid hormones exist. Signalling via adhesion receptors (ανβ3 integrins) and activation of intracellular PI-3 kinase and mitogen activated protein (MAP) kinase pathways have been proposed to account for these effects [39-41]. The precise mechanisms and the physiological implications of non-classical TH actions however remain elusive.

Besides the classic hormones T4 and T3 new data demonstrate that the rare thyroid hormone metabolite 3,5-T2 is effective in the prevention of high fat diet-induced adiposity and prevents hepatic steatosis, however, without exerting the severe side effects on the cardiac system that have been observed with T3-based treatments [42] . The vital importance of thyroid hormones for regulation of thermogenesis and for maintenance of the homeostasis of the mitochondrial energy metabolism has long been established. However, the functional interactions between the activities of uncoupling proteins (UCP) which are triggered by T3 and catecholamines affecting brown adipose tissue (BAT) as well as skeletal muscle of the adult, provide new possibilities for therapeutic intervention in obesity that have only recently become apparent [43].

Thus in the present concept of thyroid hormone action, the cellular thyroid hormone status is defined by thyroid hormone transporters, thyroid hormone membrane receptors, thyroid hormone molecules and TAM mediated actions (Figure 1B). It is highly likely and corroborated by recent experimental results that cell type-specificity, with respect to thyroid hormone signalling, differs within and in between different organs. Thereby novel avenues are opened for refinement of the concept of organ physiology and the role of thyroid hormone derived signals including their cellular compartmentalization. Such information will complement and add to our current concepts of classical TH actions.

## Perspectives of better understanding of TH action

Epidemiology has shown unequivocally that with age the ratio of subclinical to clinically manifest thyroid disorders increases, thus thyroid disorders are a disease of the ageing population. In light of the demographic changes of our societies, improvements of human health care systems should not be limited to better management of only cardiovascular disorders, cancer, and neurodegenerative diseases. We believe that modern and future-oriented health politics and policy making institutions need to take an endocrine organ into account that has been known for decades, but is still not fully “revealed”, the thyroid gland.

The knowledge of known thyroid-specific genes, the discovery of new genes as well as the investigations of thyroid hormone- and thyronamine-mediated actions on their many target tissues will entail therapeutic potentials not only for the prevention and treatment of thyroid disorders but importantly also for major health problems that are, as yet, not well treatable with thyroid hormone molecules.

Further investigations of a seemingly familiar but frequently overlooked endocrine organ, the thyroid gland, bear hopes and promises for better management of future challenges arising from current common environmental stressors of human health such as endocrine disruptors.

## List of abbreviations used

AC: adenylylcyclase; AHD: Allan Herndon Dudley; BAT: brown adipose tissue; CH: congenital hypothyroidism; CNS: central nervous system; GPCRs: G-protein coupled receptors; MAP: mitogen activated protein; NIS: sodium-iodide symporter; RXR: retinoic X receptor; T3: 3',3,5-trioodothyronine; T4: thyroxine; TAAR: trace amine associated receptor; TAMs: thyronamines; Tg: thyroglobulin; TH: thyroid hormone; TPO: thyroid peroxidase; TR: thyroid hormone receptor; TSH: thyroid stimulating hormone; TSHR: thyroid stimulating hormone receptor; UCP: uncoupling proteins;

## Authors’ contribution

KBr, DF and HB contributed equally, read and approved the manuscript.

## Competing interests

The authors declare that they have no competing interests.
